# The General Medical Council

**Published:** 1899-06-17

**Authors:** 


### THE GENERAL MEDICAL COUNCIL.

At the meeting on Thursday, June 8th, Mr. Horsley obtained leave to make a statement with reference to the proceedings taken in the name of the Council against the late Mr. H. K. Hunter, L.S.A. He desired to point out that the Council, as a whole, could not be held responsible for Mr. Hunter having been prosecuted for calling himself a physician, for the prosecution was undertaken in its name without the facts of the case being laid before it by the Penal Cases Committee. Mr. Brown said that when the prosecution was sanctioned the full facts of the case were certainly not placed before the Council. Members outside the Penal Cases Committee never had these facts before them. On the question of the amendment of the Medical Acts a resolution moved by Dr. Glover in favour of appointing a committee to consider the subject was opposed by Dr. Macalister, Mr. Carter, and others, on the ground that the Council should leave the initiation of this matter to persons outside. On the question of the improper issue of certificates of proficiency to midwives and to spectacle makers the recommendation of the Executive Committee was adopted to the effect that as things stand at present no action should be taken. The Council adjourned to November.

